# Protocol for non-invasive delivery of CRISPR RNPs via virus-like particles for mouse model generation

**DOI:** 10.1016/j.xpro.2026.104352

**Published:** 2026-02-04

**Authors:** Da Eun Yoon, Jiyun Yang, Tae Yeong Jeong, Jeongeun Park, Hyunji Lee, Je Kyung Seong, Kyoungmi Kim

**Affiliations:** 1 Department of Convergence Medicine, Korea University College of Medicine, Seoul 02708, Republic of Korea; 2 Department of Biomedical Sciences, Korea University College of Medicine, Seoul 02841, Republic of Korea; 3 Transgenic Core Facility, Max-Planck Institute of Biochemistry, 82152 Martinsried, Germany; 4 Laboratory for Genomic and Epigenomic Medicine, Research Institute for Veterinary Science, and BK21 PLUS Program for Creative Veterinary Science Research, College of Veterinary Medicine, Seoul National University, Seoul 08826, Republic of Korea; 5 Korea Model Animal Priority Center, Seoul National University, Seoul 08826, Republic of Korea; 6 Laboratory of Developmental Biology and Genomics, Research Institute for Veterinary Science, and BK21 PLUS Program for Creative Veterinary Science Research, College of Veterinary Medicine, Seoul National University, Seoul 08826, Republic of Korea; 7 Interdisciplinary Program in Bioinformatics and BIO MAX/N-Bio Institute, Seoul National University, Seoul 08826, Republic of Korea; 8 Interdisciplinary Program of Cancer Biology, Seoul National University Cancer Research Institute, Seoul, Republic of Korea

**Keywords:** biotechnology and bioengineering, cell biology, CRISPR, genetics, model organisms, molecular biology

## Abstract

CRISPR-virus-like particle (VLP)-induced targeted mutagenesis (CRISPR-VIM) enables genome editing in mouse embryos through non-invasive delivery of CRISPR ribonucleoproteins (RNPs) via VLPs, eliminating the need for physical manipulation and specialized expertise. We detail protocols for VLP production, titration, and treatment for diverse genome edits. This protocol is compatible with zygotes and *in vitro* fertilization (IVF)-derived embryos via simple co-culture, facilitating high-efficiency and heritable mutations with minimized off-target effects, independent of specialized equipment and conducive to reduced animal use.

For complete details on the use and execution of this protocol, please refer to Jeong et al.^1^

## Highlights

• Detailed steps for producing virus-like particles containing CRISPR ribonucleoproteins
• Instructions for safe mouse modeling without physical damage on embryos
• Guidance on efficient genetically modified mouse modeling for diverse mutations

## Abstract (editor highlights)

Publisher’s note: Undertaking any experimental protocol requires adherence to local institutional guidelines for laboratory safety and ethics.

## Graphical abstract

**Figure:**
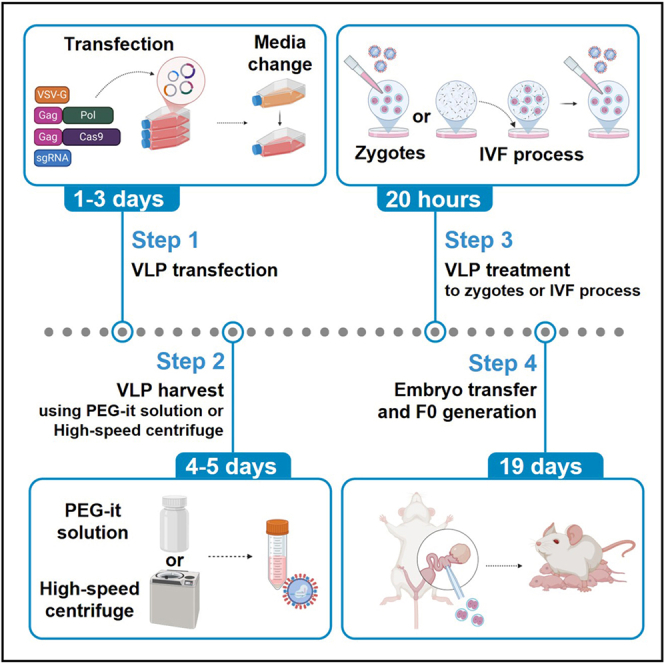

## Before you begin

Select the best sgRNA sequence optimized for your target gene before initiating virus-like particles (VLPs) producing procedure. Also, we highly recommend confirming the efficiency of VLPs in mouse embryonic stem cells (or at least other mouse cell lines if embryonic stem cells are not available) after the production of each VLP.

[Troubleshooting 1].

### Innovation

Conventional strategies for generating genetically engineered mouse models (GEMM) include microinjection and electroporation.^2^^,^^3^^,^^4^ These techniques require specialized and often expensive equipment. Especially for microinjection, precise delivery into the pronuclei of each zygote is necessary, and outcomes are highly dependent on the operator’s skill. In addition, physical manipulation increases the risk of embryo damage and may impair normal development.

In contrast, VLPs can be produced with a single transfection in laboratories equipped for standard cell culture. We introduced CRISPR-VLP-induced targeted mutagenesis (CRISPR-VIM) can induce various mutations including simple knockout, nucleotide substitution, precise knock-in in our previous publication.^1^ VLPs are simply added to the embryo culture dish, enable generation of gene-edited mice without specialized personnel and minimize physical stress on embryos.^1^

When CRISPR-ribonucleoprotein (RNP) is used, which provide immediate activity and reduced off-target risk, conventional methods such as microinjection and electroporation typically require labor-intensive in vitro protein purification.^5^^,^^6^ In the VLP workflow, RNPs are packaged directly during particle production, eliminating the need to optimize protein expression and purification for each gene-editing system.

Despite these advantages, large-fragment knock-in remains challenging using VLPs alone. In such cases, conventional approaches can be combined with VLP-mediated nuclease delivery and AAV donor co-delivery to support homology-direct repair (HDR).^7^

Still there is room to improve, VLP is broadly applicable to cells and embryos, reproducible, has high editing efficiency with low off-target, and reduces cellular stress. We believe CRISPR-VIM can let many researchers more accessible to genetically engineered animals and speed up further studies and disease treatment.^8^

### Institutional permissions

Mice should be maintained in an SPF animal facility under 12:12-h of light–dark cycle, at 20°C–26°C, and 40%–60% relative humidity. All animal use and experimental procedures must be approved by, and conducted in accordance with, the guidelines of the Institutional Animal Care and Use Committee (IACUC). We obtained approval from Seoul National University and Korea University for this project (SNU-220930-2-1, SNU-230813-1-1, KOREA-2022-0105-C2, KOREA-2022-0105).

All procedures involving cell culture and production of VLP were performed under Biosafety Level 1 (LMO No. 24–329) facility. The production and handling of AAV vectors were carried out under Biosafety Level 2 (LMO No. 16–1098) facility, following to institutional and national guidelines for recombinant DNA and viral vectors.

### Gesicle Producer 293T cell culture

**Timing: 8 days**

1. Cell thawing.
  a. Thaw a Gesicle cell vial in 37°C water bath.
  b. Add 5 mL of Gesicle culture medium and centrifuge at 300 × *g* for 3 min.
  c. Aspirate the medium and resuspend the cell pellet with 1 mL of Gesicle culture medium.
  d. Culture the thawed cells on a 100 mm collagen-coated dish and keep it at 37°C in a 5% CO_2_ incubator.
  e. Change the cell culture medium after 24 h of seeding with 10 mL of fresh Gesicle culture medium.
  f. Subculture the cells when the confluency gets approximately 80%.

***Note:*** Use the Gesicle Producer 293T cells for experiments at least after one week of stabilization period.

### Preparation of 1-cell zygotes

**Timing: 3 days**

2. Superovulation of female mouse.
  a. Inject 0.2 mL of CARD HyperOva intraperitoneally into lower abdomen of 5–8-week-old female mice 3 days prior to zygote collection.
    ***Note:*** While PMSG is widely used as an alternative, the use of HyperOva can increase the number of ovulated oocytes by approximately 3- to 5-fold. Therefore, the use of HyperOva is recommended for a high yield of embryos.^9^
    ***Note:*** We injected both hormones at 5 PM in our experiment. However, it can be adjusted according to the condition of animal facility or advice from veterinarians.
  b. Inject 7.5 I.U. of human chorionic gonadotropin (hCG) 48 h after HyperOva injection.
3. Mate each female 1:1 with a ≥10-week-old male in the evening.
4. Collect 1-cell zygotes on the following day.^10^^,^^11^
  a. Collect zygotes from oviducts of plug-positive females in M2 medium drops under a stereomicroscope.
  b. Remove cumulus cells from zygotes by incubating in 0.1% hyaluronidase, then wash five times in fresh M2 drops.

[Troubleshooting 2].

### Preparation of foster mothers

**Timing: 1 day**

5. Mate 8-week-old ICR (CD1) females with ≥12-week-old vasectomized males at 1:1 ratio on the same day as VLP treatment.
6. Separate the plug-checked ICR females from males on the other day.
7. Anesthetize the females with 1.5%–3.0% isoflurane in O_2_ (at 1 L/min flow), continuously monitoring for anesthetic depth.

## Key resources table

**Table:** 

REAGENT or RESOURCE	SOURCE	IDENTIFIER
**Antibodies**		

Anti-MuLV p30 Polyclonal Antibody; Antibody working dilution: 1:1,000	Cell Biolabs	VPK-156; 315602
Secondary Antibody, HRP Conjugate; Antibody working dilution: 1:1,000	Cell Biolabs	VPK-156; 231009
Biotin-labeled Antibody (Concentrated, 100×); Antibody working dilution: 1:100	Finetest	EU2607;E003
HRP-Streptavidin Conjugate (SABC, 100×); Antibody working dilution: 1:100	Finetest	EU2607;E034

**Bacterial and virus strains**		

Escherichia coli DH5α	Enzynomics	CP010

**Chemicals, peptides, and recombinant proteins**		

DMEM	Welgene	LM001-05
DPBS	Welgene	LB001-02
FBS, Premium, Heat Inactivated	Welgene	S101-01
Trypsin-EDTA	Welgene	LS015-10
jetPRIME	Polyplus	101000001
PEG-it™ Virus Precipitation Solution (5×)	System Biosciences	LV810A-1
Sucrose	Sigma-Aldrich	S0389-500G
Laemmli 2× concentrate sample buffer	Sigma-Aldrich	S3401
CARD HyperOva	COSMOBIO	KYD-010-EX-X5
Chorionic Gonadotropin Human	Sigma-Aldrich	CG10-1VL
Mineral Oil	Sigma-Aldrich	M5310
EmbryoMax KSOM Mouse Embryo Media	Sigma-Aldrich	MR-121-D
M2 medium	Sigma-Aldrich	M7167
Hyaluronidase from bovine testes	Sigma-Aldrich	H3884-100MG
CARD FERTIUP Preincubation Medium: PM	COSMOBIO	KYD-002-05-EX-X5
HTF (Human Tubal Fluid)	COSMOBIO	CSR-R-B070
CLEANCLE Perfusion (NaCl)	JW Pharmaceutical	N/A
Sodium hydroxide	Sigma-Aldrich	S5881-1KG
EDTA (0.5 M), pH 8.0, RNase-free	Invitrogen	AM9261
HEPES	Sigma-Aldrich	H3375-500G
Certified Molecular Biology Agarose	Bio-Rad	1613102

**Critical commercial assays**		

MuLV Core Antigen ELISA Kit	Cell Biolabs	VPK-156
FLAG-Tag (DYKDDDDK-Tag Protein) ELISA Kit	Finetest	EU2607
DNeasy Blood & Tissue Kit	QIAGEN	69506
SuperFi II PCR Master Mix	Invitrogen	12368050
KAPA HiFi HotStart plus dNTPs	Roche	7958897001
SUN PCR Blend	SUN GENETICS	SG-PT02
NucleoSpin Gel and PCR Clean-up	Macherey-Nagel	740609.50
MiSeq Reagent Micro Kit v.2	Illumina	MS-103-1002

**Deposited data**		

Targeted amplicon sequencing data		PRJNA1357287

**Experimental models: Cell lines**		

Human; Gesicle Producer 293T Cell Line	Clontech	632617

**Experimental models: Organisms/strains**		

ICR (CD1) mouse		RRID:MGI:2160272, *Mus musculus*, 8 weeks old female, ≥12 old male
C57BL/6N mouse		RRID:MGI:2159965, *Mus musculus*, 5–8 weeks old female, ≥10 old male
C57BL/6J mouse	Jackson Laboratory	RRID:IMSR JAX:000664, *Mus musculus*, 5–8 weeks old female, ≥10 old male

**Oligonucleotides**		

*TTN* sgRNA	GGTACTTCAGCAGCCTTCAC	N/A
NGS_1st_*TTN*_F	CTGCCTGCCAGAAACT ACA	N/A
NGS_1st_*TTN*_R	TCTGAACTCCAGGACT GACT	N/A
NGS_2nd_*TTN*_F	ACACTCTTTCCCTACACGACGCT CTTCCGATCTTTTGTCGGAGAAG ACTCCATTC	N/A
NGS_2nd_*TTN*_R	GTGACTGGAGTTCAGACGTGTG CTCTTCCGATCTCTGTTCACCTT TACCTTTCCTTTC	N/A
*Plin1* sgRNA	TGCCTATGAGAAGGGTGTAC	N/A
NGS_1st_*Plin1*_F	TGCCTATGAGAAGGGTGTAC	N/A
NGS_1st_ *Plin1*_R	AATAGCTCGTGAGAAGGTTGAG	N/A
NGS_2nd_ *Plin1*_F	TCAGGATGGTAGAAGATGGTAGA	N/A
NGS_2nd_ *Plin1*_R	ACACTCTTTCCCTACACGACGCTCT TCCGATCTTTCTTTGCTGACAGGAGCAG	N/A

**Recombinant DNA**		

pCMV-VSV-G	Addgene	8454
pBS-CMV-gagpol	Addgene	35614
pCMV-MMLVgag-3xNES-Cas9	Addgene	181752
pCMV-MMLVgag-3xNES-ABE8e	Addgene	181751
pCMV-AncBE4max	Addgene	112094
pRG2	Addgene	104174

**Software and algorithms**		

EUN program	daeunyoon.com	N/A
CRISPR RGEN Tools	rgenome.net	N/A
Cas-OFFinder	rgenome.net/cas-offinder/	N/A
Bionics Sanger-sequencing service	Bionics(bionicsro.co.kr)	N/A
Prism	GraphPad (graphpad.com)	N/A

**Other**		

DMi1 Inverted Microscope for Cell and Tissue Culture	Leica	DMi1
Water bath	JSR	JSWB-22T
CO2 incubator	PHCbi	MCO-170AIC
Counting chambers	Marienfeld Superior	HSU-0650010
LN2 tank	MVE	CRYOSYSTEM6000
Cryovial	SPL	43112
Cell culture dish 100 mm	SPL	20100
Conical tube 50 mL	SPL	50050
Minisart NML Surfactant-free Cellulose Acetate Standard Syringe Filter (SFCA), 0.2 μm, FM, Female Luer Lock, Male Luer Lock	Sartorius	S6534-FMOSK
60 mm cell culture dish	Sarstedt	83.3901
Aspirator tube assemblies for calibrated microcapillary pipettes	Sigma-Aldrich	A5177
Capillary glass	Harvard	BS4 30-0065
Stereoscopic microscope	Nikon	SMZ745
H401-GLASS TABLE	Okolab	H401-GLASS TABLE
CO2 incubator	PHCbi	MCO-5AC-PK
150 mm TC dish	Sarstedt	83.3903
T-75 cell culture flask	SPL	70075
100 mm cell culture dish	SPL	20100
Collagen Type I–coated cell culture dish	SPL	21100
Conical tube 40 mL	SPL	50040
1.5 mL microcentrifuge tube	SPL	60115
Kovax syringe 20 mL	KOREA VACCINE	N/A
38.5 mL Open-Top Thinwall Ultra-Clear Tube, 25×89 mm - 50Pk	Beckman Coulter	344058
0.45 μm PVDF syringe filter	Daihan Scientific	DH.Fil 3066
Vacuum bottle-top filtration system	Millipore	S2HVU02RE
Allegra X-15R centrifuge	Beckman Coulter	N/A
Centrifuge	Hanil	M15R
Optima(TM) XE-100 Ultracentrifuge	Beckman Coulter	A94516
SW 32 Ti swinging-bucket rotor	Beckman Coulter	SW32Ti
LABOSHAKER	LABOGENE	R100
Multiskan SkyHigh with touchscreen and cuvette	Thermo Scientific	A51119700C
NanoDrop One/OneC Microvolume UV-Vis Spectrophotometer	Thermo Scientific	ND-ONE
Vortex	Scientific Industries	SI-0236
Thermal cycler	Bio-Rad	T100
Heat block	Biofree	BF-40HB
Reservoir	SPL	22050
Agar tank/lid	Bioneer	A-7020-2
PowerPac Basic Power Supply	Biorad	1645050JA
Eppendorf Research plus	Eppendorf	N/A
10 μL (0.1–10 μL) Ultra G	Sorenson	23580T
200 μL (1–200 μL) Standardization	Sorenson	15720T
1000 μL (50–1250 μL)	Sorenson	31610
5 mL serological pipette	Sarstedt	86.1253.001
10 mL serological pipette	Sarstedt	86.1254.001
25 mL serological pipette	Sarstedt	86.1255.001
50 mL serological pipette	Sarstedt	86.1256.001

## Materials and equipment

### Reagent

**Table:** Gesicle culture medium

Reagent	Final concentration	Amount
Fetal bovine serum	10%	50 mL
Dulbecco’s modified eagle medium	90%	450 mL
**Total**		**500 mL**

Store at 4°C.

**Table:** Lysis buffer

Reagent	Final concentration	Amount
10M NaOH	24 mM	24 μL
250mM EDTA	0.25 mM	10 μL
Distilled water		9,966 μL
**Total**		**10 mL**

Mix or gently vortex the solution, ensuring the lysis buffer maintains pH 8.0. Store at 25°C.

**Table:** 20% (w/v) sucrose cushion buffer

Reagent	Final concentration	Amount
Sucrose	20%	20 g
Distilled water	80%	80 mL
**Total**		**100 mL**

Vortex thoroughly until sucrose is fully dissolved. Prepare fresh before use and store at 4°C.

**Table:** Hyaluronidase

Reagent	Final concentration	Amount
Hyaluronidase	0.1%	0.1 μL
DPBS	99.9%	99.9 μL
**Total**		**100 μL**

Filter-sterilize through a 0.2 μm syringe filter. Aliquot 0.7 mL per tube and store at −20°C.

• M2 dish.

Prepare five 100 μL drops of M2 medium on a 60 mm dish and cover completely with mineral oil. Warm up the dish for at least 30 min at 37°C in a 5% CO_2_ incubator before use.

• KSOM dish.

Prepare seven 30 μL drops of KSOM medium on a 60 mm dish and partially cover with mineral oil to fix the position of drops. Add 20 μL additional KSOM to each drop, then cover completely with mineral oil. Warm it up for at least 30 min at 37°C in a 5% CO_2_ incubator.

• Sperm dish.

Prepare a 100 μL drop of CARD Fertiup PM in the center of a 60 mm dish and cover completely with mineral oil. Warm up for at least 30 min at 37°C in a 5% CO_2_ incubator.

• Fertilization dish.

Prepare a single 100 μL drop of HTF medium on a 60 mm dish and cover completely with mineral oil. Warm it up for at least 30 min at 37°C in a 5% CO_2_ incubator.

• Wash & Culture dish.

Prepare four 100 μL drops of HTF medium on a 60 mm dish and completely cover with mineral oil. Warm the dish up for at least 30 min at 37°C in a 5% CO_2_ incubator.

### Equipment setup

• Ultracentrifuge.

**CRITICAL:** Pre-cool down the centrifuge to 4°C prior to use.

For Beckman Coulter ultracentrifuges, centrifuge samples at 115,151 × *g* for ≥2 h at 4°C to pellet VLPs.

• Microplate reader for ELISA.

Set the optical density (OD) measurement to 450 nm including a 10 s shaking step immediately before measurement to ensure sample homogeneity.

## Step-by-step method details

### Production of VLPs

**Timing: 5 days**

**Figure 1:**
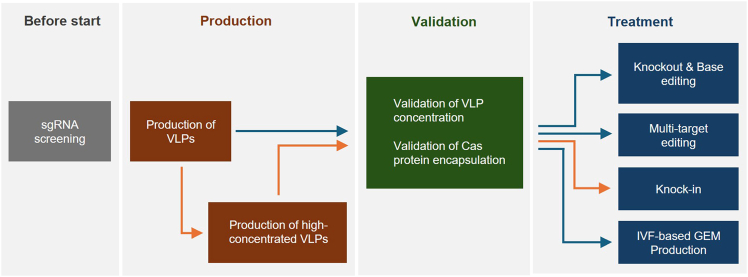
Overview of the procedure The procedure is divided into VLP production, validation, and treatment for specific applications. Prior to beginning, sgRNA should be optimized in mouse cell lines or embryonic stem cells. Efficient knock-in requires highly concentrated VLP preparation, as indicated by the orange line. The blue line denotes experiments using the VLP concentration step with PEG-it precipitation solution.

Here, we detail the process for producing VLPs containing CRISPR RNPs (Figure 1). High-quality and high-yield VLP production is essential, as it directly influences editing efficiency. We recommend using the reagents and parameters specified in this protocol. Additionally, we describe a method for generating highly concentrated VLP preparations via ultracentrifugation. For HDR-mediated knock-in applications, further concentration (>1 × 10^9^ VLPs/μL) is required. These preparations may also be used for other editing applications (see E, F, and H), though such concentration is unnecessary for those steps.

**Figure 2:**
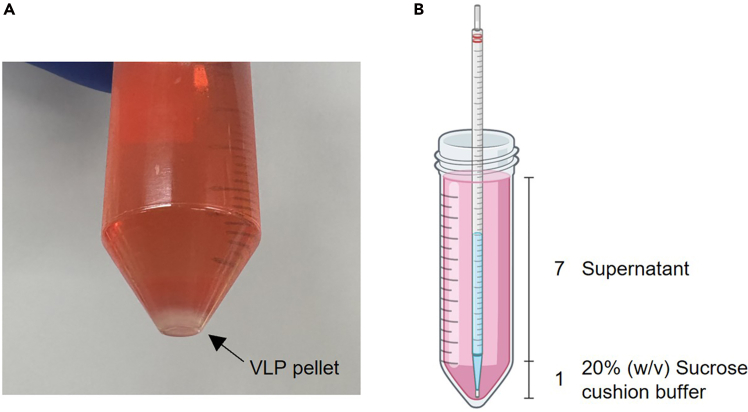
Concentration of VLPs using PEG-it™ Virus Precipitation Solution and Ultracentrifugation (A) VLP pellet collected from one T-75 cell culture flask after 16h of incubation with PEG-it™ Virus Precipitation Solution and subsequent centrifugation at 1,500 × g, 4°C, 30 min. (B) Addition of 20% (w/v) sucrose cushion buffer under VLP-containing filtered supernatant.

1. Gesicle cell seeding.
  a. Harvest Gesicle cells from culture plates at approximately 80% confluency.
    ***Note:*** A fully confluent 150 mm culture dish of Gesicle cells is generally sufficient to seed 7–8 T-75 cell culture flasks.
    **CRITICAL:** Maintain the passage number of Gesicle Producer 293T cells below 20 to ensure efficient and consistent VLP production.
  b. Seed 5 × 10^6^ Gesicle cells in a T-75 cell culture flask containing 10 mL Gesicle culture medium.
    **CRITICAL:** Distribute Gesicle cells evenly across the flask for optimal growth.
    ***Note:*** This protocol describes a single T-75 cell culture flask, but multiple flasks may be prepared as needed. For high-concentration VLP production (>1 × 10^9^ VLPs/μL), we recommend ≥10 T-75 flasks for knock-in (see ‘production of high-concentration VLPs’).
  c. Culture the cells for 16–24 h at 37°C in a 5% CO_2_ incubator.
2. Transfect VLP-expressing plasmids to Gesicle.
  ***Note:*** For details on the VLP-expressing plasmids used, see Table S1.
  a. On the following day (16–24 h post-seeding), prepare the plasmid mixture for transfection in a 1.5 mL tube (Table 1).^12^
  b. Add 500 μL JetPrime® buffer to the plasmid mixture and vortex for 3 s.
    ***Note:*** Store JetPrime® buffer refrigerated (4°C) until use.
    **CRITICAL:** Vortex the plasmid mixture to avoid DNA aggregation.
  c. Add 20 μL JetPrime® transfection reagent to the same tube and vortex for 3 s.
    ***Note:*** Store JetPrime® transfection reagent refrigerated (4°C) until use.
  d. Incubate the plasmid–JetPrime® mixture at 25°C for 10 min to allow complex formation.
  e. Gently add the mixture to the cultured Gesicle cells in the T-75 flask, tilt the flask to mix gently. Incubate at 37°C, 5% CO_2_.
    **CRITICAL:** Do not pipette the JetPrime® transfection mixture directly onto the cells to avoid localized toxicity.
3. Medium change.
  a. After 24 h, gently remove the culture medium and replace with 20 mL fresh Gesicle culture medium.
    **CRITICAL:** Avoid excessive detachment of Gesicle Producer 293T cells during medium change; During medium changes, we recommend gently tilting the flask or dish, removing medium slowly from the edge, and adding fresh medium along the opposite side of the wall with the cells to reduce mechanical shear stress. Users should also avoid tapping the flask, vigorous swirling, or dispensing medium directly onto the cell layer, as these actions can disrupt the monolayer.
  b. Incubate at 37°C, 5% CO_2_.
4. Harvest the VLPs and concentrate them.
  a. After 48 h post-transfection, collect the culture supernatant and filter through a 0.45 μm PVDF syringe filter or a vacuum bottle-top filter to remove cell debris.
    ***Note:*** Filter clogging or slow filtration may indicate poor VLP quality.
    [Troubleshooting 3 and 4].
    ∗Proceed to ‘production of high-concentration VLPs’ if higher-concentration VLPs (>1 × 10^9^ VLPs/μL) are required.
  b. Collect the filtrate (20 mL) and add 5 mL PEG-it™ Virus Precipitation Solution (5×). Gently invert 5 – 8 times to mix it thoroughly and store at 4°C for ≥16 h.
    **CRITICAL:** Mix thoroughly until the solution is clear.
  c. Move the mixture to 50 mL conical tube and centrifuge them on the following day at 1,500 × *g*, 4°C, for 30 min to pellet the VLPs using SX4750 rotor of Allegra X-15R centrifuge (Figure 2A).
5. Carefully remove the supernatant and gently resuspend the pellet in at least 100 μL Opti-MEM, gently mix it until it’s fully dissolved.

**Table 1:** Transfection mixture for VLP production

Plasmid	Quantity
VSV-G	400 ng
pBS-CMV-gagpol	3,375 ng
pCMV-MMLVgag-3xNES-Cas9 (or ABE or CBE)	1,125 ng
pRG2 (containing your selected target sequence)	4,400 ng

[Troubleshooting 6] Do not use DPBS. Using DPBS for VLP resuspension may cause precipitation under embryo culture conditions.

6. Aliquot the final VLP solution into suitable tubes and store at −80°C until use.

***Note:*** Avoid repeated freeze–thaw cycles; thaw each aliquot only once.

***Note:*** Reserve 1 μL of the final VLP solution for titration.

**Pause point:** Frozen VLPs remain usable for up to 12 months if properly stored.

### Production of high-concentration VLPs

**Timing: 6 h**

7. Follow steps 1–4a from ‘production of VLPs’.

***Note:*** ‘production of high-concentration VLPs’ assumes the use of 10 T-75 cell culture flasks for high-yield production.

8. Concentrate produced VLPs using ultracentrifugation.
  a. Transfer the filtered supernatant into 40 mL conical tubes. Reserve 1/8 of the total tube volume for subsequent sucrose addition.
    ***Note:*** Use centrifuge-compatible tubes suited to the selected rotor.
  b. Carefully add 20% (w/v) sucrose cushion buffer, equal to 1/7 of the supernatant, to the bottom of each tube using a serological pipette, ensuring a distinct layer beneath the supernatant (Figure 2B).
    **CRITICAL:** Avoid mixing the sucrose layer with the supernatant to promote efficient VLP pelleting.
  c. Centrifuge samples at 4°C for at least 1 h at a minimum force of 115,151 × *g*.
    **CRITICAL:** Use swing rotor for filtering and concentration.
    ***Note:*** Ultracentrifugation is performed using a SW 32 Ti swinging-bucket rotor (Beckman Coulter) at 115,151 × g (26,000 rpm).
  d. Confirm visible pellet formation, then carefully remove supernatants.
    **CRITICAL:** If the pellet is too small, promptly discard the supernatant after centrifugation to minimize VLP loss.
    ***Note:*** For visible pellet formation, use supernatant from more than two T-75 flasks; smaller volumes may not yield sufficient pellets.
  e. Overlay fresh filtered supernatant from another flask directly onto the pellet to accumulate VLPs.
  f. Repeat until all of culture supernatants have been processed and pooled onto the pellet.
  g. For the final batch, combine all VLP pellets into a new tube, using freshly filtered supernatant or Gesicle culture medium as needed. Perform a final ultracentrifugation (minimum 115,151 × g) at 4°C for ≥2 h to concentrate VLPs.
9. After centrifugation, carefully remove the supernatant and immediately add 20 μL Opti-MEM to the pellet to prevent drying.

**CRITICAL:** Use a suction system to remove residual medium and sucrose buffer completely, as residues may interfere with next steps.

**CRITICAL:** Adjust the volume of Opti-MEM for resuspension according to pellet size; ensure sufficient volume to fully dissolve the VLP pellet. Incomplete resuspension can block syringe needles or pipet tips and vary editing efficiencies.

**CRITICAL:** If the pellet does not dissolve completely, add serial 10 μL Opti-MEM until fully resuspends.

**CRITICAL:** Avoid placing the pipette tip near the pellet during resuspension to prevent particle loss.

10. Aliquot the final VLP solution into suitable tubes and store at −80°C until use.

***Note:*** Avoid repeated freeze–thaw cycles; thaw each aliquot only once.

***Note:*** Reserve 1 μL of the final VLP solution for p30 titration.

**Pause point:** Frozen VLPs remain usable for up to 12 months if properly stored.

### Validation of VLP concentration using QuickTiter MuLV Core Antigen ELISA Kit (MuLV p30)

**Timing: 6 h**

In this part, we describe validation of VLP particle number and quantification of Cas proteins per VLP particle (Figure 1). Both parameters are measured by ELISA. We outlined this calculation based on previously published methods and data.^1^^,^^12^^,^^13^

11. Prepare samples for ELISA.
  a. Thaw 1 μL of the final VLP solution and add 999 μL Assay Diluent (provided in the ELISA kit).
  b. Prepare a serial dilution of the VLP sample.
    **CRITICAL:** For VLPs concentrated with PEG-it™ Virus Precipitation Solution (5×) from ‘Production of VLPs’, prepare 1/10 serial dilutions from 1:1,000 to 1:100,000.
    **CRITICAL:** For VLPs concentrated by ultracentrifugation using a 20% (w/v) sucrose cushion buffer from ‘production of high-concentration VLPs’, prepare 1/10 serial dilutions ranging from 1:10,000 to 1:1,000,000.
  c. Transfer 225 μL of each diluted VLP sample and add 25 μL of Triton X-100 solution.
  d. Inactivation step: Incubate the mixture at 37°C for 30 min.
  e. Prepare p30 standards (20, 10, 5, 2.5, 1.25, 0.625, 0.313, and 0 ng/mL) by serially diluting from 2 μL of the 10 μg/mL stock standard.
  f. Add 25 μL of Triton X-100 solution to each 225 μL standard sample.
12. Measure amount of VLPs.
  a. After inactivation, add 100 μL of each diluted VLP sample and standard to individual wells of the Anti-MuLV p30 Antibody Coated Plate.
    ***Note:*** All samples, blanks, and standards must be assayed in duplicate to ensure data reliability.
  b. Cover the plate and incubate on a platform orbital shaker at 20 rpm for 2 h at 25°C.
  c. Aspirate all liquid from the wells and wash five times with 1× Wash Buffer, taking care to avoid damaging the coating on the plate.
  d. Add 100 μL of 1:1,000 diluted Anti-MuLV p30 Polyclonal Antibody to each well.
  e. Cover and incubate on a platform orbital shaker at 20 rpm for 1 h at 25°C.
  f. Carefully aspirate all solutions from the wells and wash five times with 1× Wash Buffer.
  g. Add 100 μL of 1:1,000 diluted Secondary Antibody-HRP to each well.
  h. Cover and incubate on a platform orbital shaker at 20 rpm for 1 h at 25°C.
  i. Pre-warm Substrate Solution to 25°C before use.
  j. Carefully aspirate and wash wells five times with 1× Wash Buffer.
  k. Add 100 μL of pre-warmed Substrate Solution to each well and incubate on a platform shaker until the solution develops a blue color and a visible gradient form.
  l. Add 100 μL of Stop Solution to each well and confirm that the color changes to yellow.
  m. Measure absorbance at 450 nm using a microplate reader.
13. Calculate the number of VLPs.
  a. draw the trendline equation based on the measured standard values.
    **CRITICAL:** Ensure that the R2 value of the standard curve is ≥0.95 for accurate quantification.
  b. Substitute the sample absorbance (450 nm) into the equation to calculate values in ng/mL.
    **CRITICAL:** Discard any measurements where absorbance values exceed the assay's saturation threshold.
  c. Multiply the dilution factor.
  d. Calculate the mean value from all replicate wells for each VLP sample.
  e. Calculate VLP particles/μL.
    $$\text{VLP particles}/\mu L=\frac{p30\;\left(ng/mL\right)\times 10^{-12}}{{MW}_{p30}}\times N_{A}\times \frac{1}{p30\;molecules\;per\;VLP}\times 0.2$$
    ∗*MW*_*p*30_=30,000g/mol.
    ∗∗*N*_*A*_=6.022×10^23^ (Avogadro’s number).
    ∗∗∗p30 molecules per VLP = 1,800.
    ∗∗∗∗0.2 = intact particle correction factor^13^
    [Troubleshooting 5]
    ***Note:*** A detailed example of the calculation is provided in Table S2A. (Data indicated in gray were excluded from the calculation because their signals were comparable to saturation or background noise.)

### Validation of Cas protein encapsulation using FLAG-Tag ELISA

**Timing: 3 h**

14. Prepare samples.
  a. Mix the VLP samples with Laemmli 2× sample buffer and incubate at 95°C for 15 min to lyse the particles.
  b. Prepare sample dilutions at 1:2, 1:4, 1:8, and 1:16 using the dilution buffer provided in the FLAG-Tag (DYKDDDDK-Tag Protein) ELISA Kit. Prepare each dilution in duplicate.
    **CRITICAL:** Vortex each diluted sample thoroughly to ensure homogeneity.
  c. Prepare a lyophilized Flag-Tag protein standard curve with concentrations of 1,000, 500, 250, 125, 62.5, 31.25, 15.625, and 0 ng/mL by serial dilution using the supplied sample dilution buffer.
15. Measure the Cas protein.
  a. Wash the ELISA microplate twice with 1× Wash Buffer (350 μL per well).
    ***Note:*** Prepare 1× Wash Buffer by diluting the 20× stock solution with distilled water in advance.
  b. Dispense 50 μL of each standard and diluted sample into individual wells, then add 50 μL of 1:100 diluted Biotin-labeled antibody working solution to each well.
  c. Mix carefully and incubate the plate at 37°C for 30 min.
  d. Wash the wells three times with 350 μL of 1× Wash Buffer per wash.
  e. Add 100 μL of 1:100 diluted SABC working solution to each well and incubate as instructed.
  f. Wash the wells five times with 350 μL of 1× Wash Buffer per wash.
  g. Add 90 μL of TMB substrate to each well.
  h. Cover the plate with sealing film and incubate in the dark at 37°C for 10–20 min.
    ***Note:*** Monitor color development at regular intervals to avoid exceeding the optimal absorbance range.
  i. Add 50 μL of Stop Solution to each well.
    ***Note:*** Measure the optical density (OD) immediately after adding Stop Solution to minimize variability.
  j. Read OD values at 450 nm using a calibrated microplate reader.
16. Calculate the concentration of Cas protein.
  a. Use Curve Expert 1.3 software (provided with the ELISA kit) to generate a standard curve from the known FLAG-Tag protein concentrations.

***Note:*** Details of the calculation table are provided in Table S2B. (Data indicated in gray were excluded from the calculation because their signals were comparable to saturation or background noise.)

### Treatment of VLPs for knockout or base editing

**Timing: 20 min (+20 h incubation, transplantation on following day)**

From here, we outline the procedures for generating simple knockout, performing base editing, conducting multi-target editing (targeting more than two loci simultaneously), achieving knock-in, and applying the protocol during IVF (Figure 1). For each application, we provide detailed instructions and key precautions to ensure optimal embryo handling and maximize editing efficiency.

Approximately 20 h after VLP treatment (on the following day), 20–30 embryos in 2-cell stage are transferred to each foster mother (0.5 days post coitum, pseudopregnant females mated with vasectomized males). After birth, biopsy samples can be collected and analyzed by targeted deep sequencing on next-generation sequencing (NGS) platforms or by Sanger sequencing.

17. Prepare VLPs.
  a. Thaw the frozen VLP aliquot on ice.
  b. Gently mix the VLP solution by pipetting, then add 3 × 10^9^ VLP particles to 50 μL KSOM medium in a pre-warmed dish. Incubate the dish in a 5% CO_2_, 37°C incubator for 10 min.

**CRITICAL:** Remove an equivalent volume of KSOM medium before adding VLPs.

**CRITICAL:** Keep the VLP volume below 20% of the total culture medium volume.

[Troubleshooting 6].

18. Collect zygotes from M2 medium (see ‘preparation 1-cell zygotes’) and wash three times in fresh KSOM drops.
19. Co-culture VLPs with embryos.
  a. Transfer up to 40 zygotes into each KSOM drop containing VLPs.
  b. Incubate the embryo dish in 5% CO_2_, 37°C incubator and culture the zygotes for 20 h.
  c. On the following day, select only 2-cell stage embryos and transfer 20–30 embryos into the oviduct of each 0.5 dpc pseudopregnant foster mother.

[Troubleshooting 7].

### Treatment of VLPs for multi-target editing

**Timing: 20 min (+20 h incubation, transplantation on following day)**

20. Prepare VLPs.
  a. Thaw the frozen VLP aliquot on ice.
  b. Gently mix the VLP solution by pipetting, then add the appropriate volume of VLPs to achieve total of 3 × 10^9^ particles in the KSOM medium. Incubate the dish in a 5% CO_2_, 37°C incubator for 10 min.

**CRITICAL:** Remove an equivalent volume of KSOM medium before adding VLPs.

**CRITICAL:** Keep the VLP volume below 20% of the total culture medium volume.

**CRITICAL:** For two target sites, use 1.5 × 10^9^ particles of each VLP; for three targets, use 1 × 10^9^ particles of each VLP.

[Troubleshooting 6].

21. Follow the procedure described in steps 18–19.

[Troubleshooting 7].

### Treatment of VLPs for knockin

**Timing: 20 min (+20 h incubation, transplantation on following day)**

22. Prepare self-complementary adeno-associated virus (scAAV) containing the desired insert sequence at a concentration of ≥1 × 10^12^ genome copies (GC)/mL.

***Note:*** scAAV exhibits higher stability and editing efficiency compared to single-stranded AAV (ssAAV). Serotype 6 is strongly recommended for embryo editing.^1^

***Note:*** Excessively high amount of scAAV can adversely affect embryo development and survival rates.

***Note:*** The AAV donor vector is constructed using the pscAAV-CMV-GFP (Addgene, #32396) plasmid as a backbone.

[Troubleshooting 6].

23. Follow steps E17 to prepare VLPs in KSOM drops.
24. Co-delivery of scAAV and VLP to embryos.
  a. Gently add 1 × 10^9^ GCs of scAAV to the same KSOM drop containing VLPs.
  b. Proceed with steps 18–19 for embryo co-culture, incubation, and transfer to foster mothers.

[Troubleshooting 7].

### Treatment of VLPs for *in vitro* fertilization

**Timing: 7 h (+20 h incubation, transplantation on following day)**

25. Follow the procedure described in step: ‘preparation 1-cell zygotes, step 2’ for superovulation induction in female mice.
26. On the following day, collect sperm from 11–16-week-old male mice into the sperm dish, 30 min before oocyte collection.
27. Collect oocytes from super ovulated female mice and place them in the fertilization dish containing HTF medium.
28. *In vitro* fertilization.
  a. Add activated sperm to the fertilization dish containing the oocytes.
  b. Incubate the dish in a 37°C, 5% CO_2_ incubator for 4 h to allow in vitro fertilization.
  c. Wash the in vitro fertilized eggs three times in each drop of the wash & culture dish to remove residual sperm.
29. VLP co-culturing with in vitro fertilized embryos.
  a. Thaw the frozen VLP aliquot on ice.
  b. Remove an equivalent volume of HTF medium from the final drop in the IVF wash dish and gently add 3 × 10^9^ VLPs, adjusting the final volume to 50 μL.
    ***Note:*** Ensure that the final drop volume does not exceed 50 μL to maintain proper embryo exposure to VLPs.
    [Troubleshooting 6].
  c. Proceed with steps 18–19 for co-culture, incubation, and embryo transfer.
    [Troubleshooting 7].

### Genotyping of on/off-target analysis

**Timing: 5 h (+1 day for NGS analysis)**

30. Design PCR primers to confirm editing efficiency.

***Note:*** Off-target can be analyzed by Whole Genome Sequencing or amplifying predicted off-target site using Cas-OFFinder.

***Note:*** For Cas-OFFinder prediction, off-target candidate sites allowing up to 3 mismatches and design specific primers for each site.^14^

31. Incubate embryos or mouse tissues in lysis buffer (25 mM NaOH and 0.2 mM EDTA in D.W.) at 98°C for 20–30 min for lysis in high pH (>8.0).
32. Add 0.5 M HEPES to neutralize the lysate. (pH 6.0–7.0).
33. Perform PCR using the lysate as a template.

***Note:*** For next-generation sequencing (NGS) analysis, conduct a 3-step nested PCR to incorporate adaptors and index.

***Note:*** Use high-fidelity polymerase to minimize PCR-derived errors.

34. Purify the PCR amplicons using PCR clean-up kit and analyze the editing outcomes around the target site using Sanger sequencing or NGS.

***Note:*** NGS data analysis tools are available online (e.g. EUN, RGEN tools).

***Note:*** To account for background sequencing errors, set a cut-off value of 1% for off-target detection.

***Note:*** For NGS analysis, ensure a sequencing depth of at least 5,000 reads per amplicon to reliably detect editing frequency off-target mutations.

## Expected outcomes

### VLP production and titration

Absorbance values at 450 nm from the ELISA assay should be screened to exclude readings exceeding the saturation threshold or approaching background levels. Using the standard curve equation, convert absorbance to ng/mL, adjusting for the fact that only 20% of the total detected p30 protein is fully incorporated into VLPs. Each VLP contains approximately 1,800 p30 molecules, allowing calculation of final VLP concentrations.^1^^,^^12^^,^^13^

### PEG-based concentration vs. ultracentrifuge-based concentration

Quantification of VLPs using the MuLV Core Antigen ELISA Kit showed that PEG-it^TM^-based precipitation typically yields approximately 1–3 × 10^8^ VLPs/μL, whereas ultracentrifugation with a sucrose cushion produces more concentrated VLP stocks ranging from 1 × 10^9^ to 1 × 10^10^ VLPs/μL.

### Embryo editing efficiency and developmental rate

**Figure 3:**
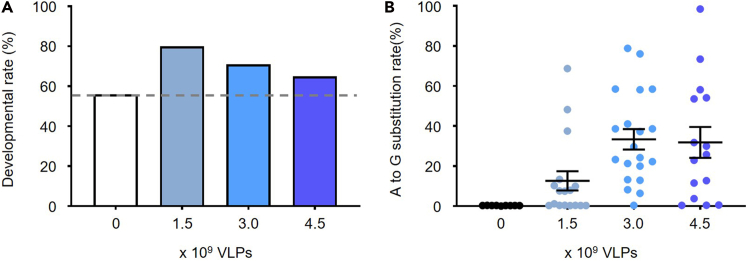
Editing efficiency and embryonic developmental rate after VLP treatment (A) Morula and blastocyst stage developmental rates from 2-cell embryos treated with different amounts of VLPs. (B) A-to-G substitution efficiency in embryos following treatment with varying VLP doses. Each dot represents one embryo (0; n=9, 1.5; n=17, 3.0; n=20, 4.5; n=15) and data are presented as scatter dot plots with mean ± SEM.

To optimize VLP dosage, we compared treatment of zygote embryos with 1.5, 3.0, or 4.5 × 10^9^ VLPs per 50 μL drop (Figure 3). Genome editing efficiency saturated at 3.0 × 10^9^ VLPs, with no further increase beyond this dose (Figure 3A). When embryos were cultured to the morula and blastocyst stages, survival rates were approximately 71% for 3.0 × 10^9^ VLPs and 65% for 4.5 × 10^9^ VLPs (Figure 3B; Table S3). Based on these findings, we recommend treating embryos with 3.0 × 10^9^ VLPs per drop of KSOM medium to achieve high editing efficiency while maintaining optimal embryo viability.

## Limitations

Compared with conventional microinjection or electroporation methods, our VLP-based delivery system offers several key advantages. It reduces the risk of physical damage to embryos and enables more consistent developmental outcomes. The workflow is technically simple and broadly accessible, relying only on transient transfection of producer cells and embryo co-culture. As demonstrated previously, it is compatible with various CRISPR tools, such as SpCas9, base editors, and supports multi-target editing. The protocol also enables germline transmission of mutations and is effective with embryos obtained from either natural mating or IVF. In addition, this system is readily scalable and suitable for high-throughput applications, with potential for large-scale embryo editing experiments. VLPs can be aliquoted and stored at −80°C, retaining editing activity after thawing and thereby allowing flexibility in experimental scheduling. However, the efficiency of large, HDR-based knock-ins remains limited, so further optimization may be required for large-fragment insertions, particularly when the donor DNA size exceeds the packaging capacity of AAV vectors. Currently, the protocol has been validated in mice, with ongoing efforts to extend its applicability to other species.

## Troubleshooting

### Problem 1

Low editing efficiency.

### Potential solution

Optimize your target sgRNA sequence in embryo cells or embryonic stem cells before making a new mouse line.

### Problem 2

Less number of oocytes or zygotes.

### Potential solution

Adjust the hormone injection time depending on the animal house rules.

### Problem 3

Color change of the culture medium in yellow.

### Potential solution

Contamination: Prepare a fresh plasmid preparation step and take caution during handling of VLPs production.

### Problem 4

Clogging of 0.45 μm PVDF filter.

### Potential solution

• Over-detachment of Gesicle Producer 293T cells: Before filtration, centrifuge supernatant at 1,500 × *g* for 5 min to remove cell debris.
• Contamination: Check the quality of transfection reagent and plasmid or thaw a fresh cell stock if necessary.

### Problem 5

No or less yield of VLP.

### Potential solution

• Producer cell: Use Gesicle cells from Clontech. Not normal HEK293T cells **[TS1].**
• Culture plate: It is better to use T-75 cell culture Flask than 100 mm culture dish.
• Poor culture medium quality: The culture medium must be replaced with fresh medium 24 h after transfection **[TS2].**
• Over-detachment of Gesicle cells: Because Gesicle cells detach easily, gentle handling is essential during plasmid transfection and media replacement.
• Not enough plate number: Manufacture multiple flasks on a single production batch.
• High concentration & minimum volume: Produce at least ten flasks (200 mL of culture media) for more than 1 × 10^9^ VLPs/μl.
• Sucrose ratio: Use exact 20% (w/v) sucrose cushion buffer for making layers when you do high centrifugation.
• Embryo number in VLP containing drop: Do not put over 40 embryos in a drop. Prepare multiple drops when you have more than 40 embryos.

#### [TS1] Comparison of producer cell lines

**Figure 4:**
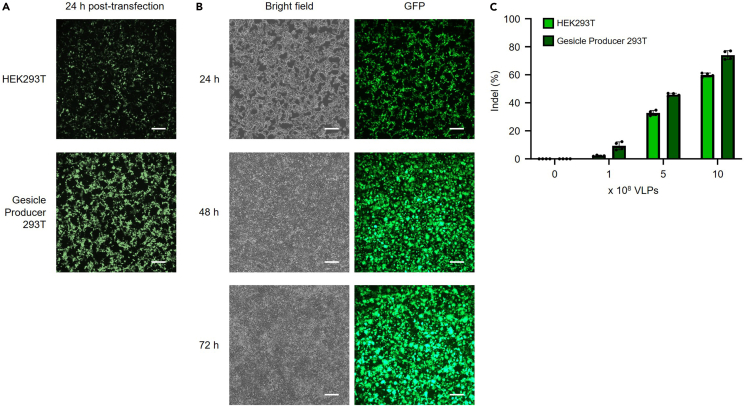
Troubleshooting images for VLP-producing cell lines (A) Fluorescence images of GFP expression in HEK293T and Gesicle cells 24 h post-transfection during VLP production. (B) Time-dependent changes in GFP expression in Gesicle cells. For (A) and (B), an sgRNA vector expressing EGFP under the hPGK promoter was used. Scale bar: 200 μm. (C) Comparison of gene editing efficiencies of VLPs produced in HEK293T and Gesicle cells. Each dot represents individual biological experiments (n = 4) and bar graph was presented with mean ± SD.

Previous attempts to produce VLPs have typically used HEK293T cells. Our data demonstrate that Gesicle cells enable significantly higher plasmid expression and more efficient VLP production than HEK293T cells (Figure 4A). Notably, plasmid expression in Gesicle cells increases over time, resulting in greater VLP output at later time points (Figure 4B). We observed markedly higher indel frequencies in cells treated with VLPs generated from Gesicle cells (Figure 4C; Table S4). These findings indicate that Gesicle-derived particles possess superior functional quality. Accordingly, we recommend using Gesicle cells and harvesting VLP-containing supernatant 48 h after medium change to maximize both particle yield and editing efficiency.

#### [TS2] The importance of medium replacement

**Figure 5:**
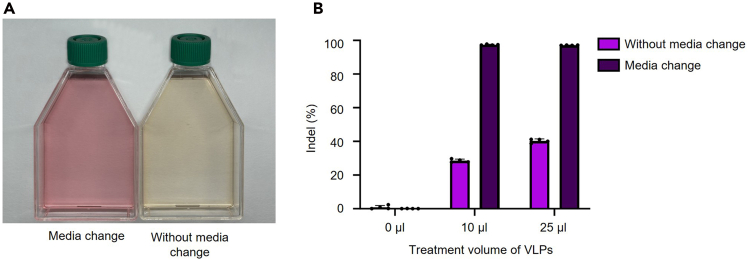
Troubleshooting images for medium replacement (A) Color differences observed in Gesicle culture medium at 72 h post-transfection. Left: Medium exchanged 24 h after transfection with fresh culture medium, Right: Medium was not exchanged 24 h after transfection. (B) Indel efficiency of produced VLPs (medium changed or not at 24 h post-transfection). Data are shown as bar with dots of each biological experiment (n = 4) and mean ± SD.

The pH and quality of the culture medium are key determinants of VLP quality and function. We evaluated outcomes when maintaining the original medium versus replacing it 24 h post-transfection in T-75 cell culture flasks (Figure 5A). Medium replacement produced cleaner supernatants and improved VLP quality, whereas omission of this step increased background contaminants and diminished VLP functional activity (Figure 5B; Table S5). Based on these results, we recommend replacing the culture medium 24 h after transfection to maximize VLP quality.

### Problem 6

Precipitates in final product of VLP in embryo culture.

### Potential solution

Use Opti-MEM for the last step of resuspension, not PBS.

### Problem 7

Low chance of pregnant foster mother number/Small litter number.

### Potential solution

Transplant more 2-cell embryos to a foster mother.

## Resource availability

### Lead contact

Further information and requests for resources and reagents should be directed to and will be fulfilled by the lead contact, Kyoungmi Kim (kyoungmi_kim@snu.ac.kr).

### Technical contact

Technical questions on executing this protocol should be directed to and will be answered by the technical contacts, Da Eun Yoon (ekidmes@naver.com), Jiyun Yang (yunej7132@naver.com), and Tae Yeong Jeong (j9t2y9@naver.com).

### Materials availability

This study did not generate new unique reagents.

### Data and code availability

The accession number for the next-generation sequencing data reported in this paper is NCBI Sequence Read Archive (SRA): PRJNA1357287 (SRP640598).

## Acknowledgments

This work was supported by the National Research Foundation of Korea (NRF) grant funded by the Korea government (RS-2023-00261905, RS-2023-NR077033, RS-2024-00441068, and RS-2025-02216523). This work was also supported by the New Faculty Startup Fund and Creative-Pioneering Researchers Program from Seoul National University. We sincerely thank Jang Hyeon Lee and Yerim Lee for their valuable assistance with the manuscript review and VLP production. The illustrations were generated with the assistance of BioRender.

## Author contributions

D.E.Y., J.Y., T.Y.J., and K.K. designed and wrote the manuscript. J.P., H.L., and J.K.S. discussed the results and commented on the manuscript. K.K. supervised the paper.

## Declaration of interests

D.E.Y., T.Y.J., J.K.S., and K.K. have filed a patent application for “Generation of genetically engineered animals by CRISPR-VLP system,” which was applied to the Korean Intellectual Property Office (10-2024-0046182) and the Patent Cooperation Treaty (PCT/KR2025/002806).

## Supplemental Information

Table S1. Coding sequence of VLP-producing plasmid, related to step 2

Table S2. Calculation of p30 and FLAG, related to step 11-16

Table S3. A-to-G conversion efficiencies in mouse embryos, related to expected outcomes, related to Figure 3

Table S4. Comparison of editing efficiency across VLP-producing cell lines, related to troubleshooting 5

Table S5. Assessment of VLP efficiency based on media replacement, related to troubleshooting 5
